# Three Challenges for AI-Assisted Decision-Making

**DOI:** 10.1177/17456916231181102

**Published:** 2023-07-13

**Authors:** Mark Steyvers, Aakriti Kumar

**Affiliations:** Department of Cognitive Sciences, University of California, Irvine

**Keywords:** AI-assisted decision-making, human-AI collaboration, advice taking, mental models, human-computer interaction

## Abstract

Artificial intelligence (AI) has the potential to improve human decision-making by providing decision recommendations and problem-relevant information to assist human decision-makers. However, the full realization of the potential of human–AI collaboration continues to face several challenges. First, the conditions that support complementarity (i.e., situations in which the performance of a human with AI assistance exceeds the performance of an unassisted human or the AI in isolation) must be understood. This task requires humans to be able to recognize situations in which the AI should be leveraged and to develop new AI systems that can learn to complement the human decision-maker. Second, human mental models of the AI, which contain both expectations of the AI and reliance strategies, must be accurately assessed. Third, the effects of different design choices for human-AI interaction must be understood, including both the timing of AI assistance and the amount of model information that should be presented to the human decision-maker to avoid cognitive overload and ineffective reliance strategies. In response to each of these three challenges, we present an interdisciplinary perspective based on recent empirical and theoretical findings and discuss new research directions.

Over the past decade, artificial intelligence (AI) has been increasingly leveraged to assist humans in various domains. Simple tasks are now automated through digital AI assistants such as Siri and Alexa. People lean on advanced driver-assistance systems to improve their driving experience. Recommender systems on media platforms supply personalized playlists that include both users’ favorite content and new content they may enjoy. This integration of AI into people’s daily lives holds the promise of saving human effort, avoiding blind spots of human decisions, and potentially saving lives. However, many challenges plague human–AI collaboration. Deployed AI systems have faced public scrutiny for propagating systemic biases (Gebru, 2020; Raji & Buolamwini, 2019), poorly generalizing to examples outside of their training data (Shen et al., 2021), and optimizing for user engagement at the cost of users’ well-being. These problems stem from the lack of alignment of these AI systems with the goals and values of the human users (Christian, 2020; Gabriel, 2020). To create AI that aligns with human values and expectations, researchers need to specify utility functions that reflect human values, which remains a challenge. In its current form, AI cannot independently make decisions that are accurate, acceptable, and fair to humans. Therefore, it is critical to take into account a human decision-maker’s (DM) expertise and feedback in addition to an AI’s computation when making decisions.

In this article, we focus on a narrow set of challenges related to “AI-assisted decision-making” in which the AI provides assistance in the form of predictions and/or explanations to a human DM who makes the final decision. For instance, AI systems have been developed to assist experts in clinical diagnosis (Rajpurkar et al., 2020; Sayres et al., 2019), financial (Bussmann et al., 2021), and judicial (Grgić-Hlača et al., 2019) decision-making, and forecasting (Benjamin et al., 2023). A growing body of literature on AI-assisted decision-making has emerged, spanning several disciplines and areas of study, including human-computer interaction, AI and machine learning, and psychology (Lai et al., 2021).

Rather than providing a systematic review of this literature, we synthesize some of the insights that have emerged, focusing exclusively on the performance-related aspects of AI-assisted decision-making. Specifically, we examine three main challenges that affect decision accuracy when an AI assists a human DM on independent tasks. First, we discuss the need to develop AI that can complement the abilities of a human DM. When a proficient AI assistant is integrated into the decision-making process, it is essential for the human to possess a good understanding of the AI’s capabilities and constraints. Second, we underscore the importance of precise human mental models of the AI. AI assistance can reach its full potential only if individuals know how to use this support to enhance the performance of human–AI teams. Third, we discuss the challenge of developing effective methods of human–AI interaction in different workflows in which humans and AI work collaboratively. This involves determining when to present AI assistance and what information to present and considering the need for AI systems to adapt to human cognitive limitations. We argue that cognitive modeling is useful for understanding the barriers facing the effective use of AI information. Table 1 includes real-world examples of these three challenges, including clinical decision support, credit assessment, and advanced driver-assistance systems, in which AI-assisted decision-making is slowly becoming the norm.

**Table 1.** Real-World Examples of AI-Assisted Decision-Making and the Associated Challenges Examined in This Article

Examples	Challenge 1: AI complements human DM’s abilities	Challenge 2: Human DM understands AI capabilities	Challenge 3: Effective interaction between AI and human DM
Clinical decision-support systems	AI identifies instances that doctors may miss	Doctors know which subpopulations and diseases the AI is good at predicting	Provide doctors appropriate explanations and exploration tools
Credit risk-assessment systems	AI assistance allows quickly leveraging vast amounts of data	Human DMs identify sensitive cases and bring subjectivity to risk assessment	Allow human DMs to investigate feature-based counterfactuals
Advanced driver-assistance systems	AI driving reduces cognitive and physical load on drivers	Drivers know when they must take over control from the AI assistant	Design appropriate nudges to indicate confidence of AI assistant

Note: AI = artificial intelligence; DM = decision maker.

In relation to each of these challenges, we delve into ongoing interdisciplinary research in both empirical and theoretical contexts. Furthermore, we propose directions for future research that can help address these challenges more effectively.

## Challenge 1: Understanding the Determinants of Human–AI Complementarity

Humans frequently deliberate on problems in groups of two or more and can achieve performances higher than any single individual in the group (Kameda et al., 2022). Previous work investigating collaborative work between humans suggests that performance improvements are often due to complementary divisions of labor between group members (Stasser & Abele, 2020). The introduction of AI into previously human-only workflows is motivated by this objective of improving decision accuracy by leveraging the complementary strengths of the human DM and the AI. At a minimum, we expect humans aided by AI to perform better (or at least not worse) than humans who make decisions unaided. Many studies have been able to achieve this benchmark primarily because they involved situations in which the human was offered AI advice by an AI that exhibited higher accuracy than could be produced through human-only performance (Vodrahalli et al., 2022; Y. Zhang et al., 2020). In this case, the human could follow the simple heuristic of always following the AI’s advice to improve performance. However, this situation raises the question of why a human should be involved at all in the decision-making process in the absence of relevant ethical and legal considerations. A more compelling scenario arises when AI-assisted performance exceeds not only unassisted human performance but also the performance of the AI itself. This situation is known as “complementarity” (Bansal, Wu, et al., 2021; Steyvers et al., 2022) and indicates that human–AI performance is better than the performance of a human or AI in isolation. Although some studies have shown promising results regarding situations in which the combined performance of the human and the AI exceeds the performance of the AI or the human in isolation (Bansal, Wu, et al., 2021; Tejeda et al., 2022), other studies have shown that the human DM does not contribute to such performance and that the AI acting by itself leads to better performance (Feng & Boyd-Graber, 2019; Green & Chen, 2019; Lai & Tan, 2019; Y. Zhang et al., 2020).

To understand the conditions under which AI-augmented decision-making leads to complementary performance, it is helpful to distinguish between two different reasons for the potential failure to achieve complementarity. First, it is important to understand what sorts of information the human DM and the AI can contribute independently and whether this information can (theoretically) lead to complementarity. For example, Steyvers et al. (2022) identified some general conditions for complementarity in the domain of classification. The investigation focused on pairs of classifiers: human–human, hybrid human–AI, and AI–AI (specifically, two different machine classifiers) pairs. The findings indicated that hybrid human–AI pairs, which combine human predictions with varying degrees of accurate AI predictions, can surpass the performance of either human–human or AI–AI pairs. This superior performance is achieved as long as the disparity in accuracy between human and AI predictions remains beneath a specific threshold. This threshold is contingent on the latent correlation, which signifies the level of independence between human and AI predictions. When the correlation between human and AI predictions is low, merging the predictions of a highly accurate AI with those of a less accurate human (or vice versa) can still result in superior performance than that achieved by either a pair of humans or a pair of AIs. Likewise, research on decision-making in human groups has shown that statistical combinations of individual predictions can lead to accurate group performance when the group is composed of cognitively diverse individuals, producing uncorrelated predictions (Davis-Stober et al., 2015; Hong & Page, 2004).

In the case of AI-augmented decisions, the final prediction is not created using statistical means but, rather, is the result of a cognitive process within the human DMs, who must combine the AI prediction with their own independent information. Ideally, humans rely on the AI in the context of problems in which the AI is more accurate and rely on their own judgment when the AI is less accurate. To ensure appropriate reliance, it is crucial to help humans correctly identify regions of complementary ability of the AI. This may be done by providing AI confidence or explanations to help humans better understand the AI’s decision. When the nature of complementarity is easy for people to identify, people can make appropriate reliance decisions (Q. Zhang et al., 2022). However, it is not entirely clear whether human DMs can exploit the potential for complementarity more generally. Therefore, complementarity can fail because the DM is unable to achieve it because of suboptimal reliance decisions despite the fact that the potential for complementarity existed.

Alternatively, complementarity can fail because the potential for complementarity never existed from a statistical perspective (e.g., the performance difference between humans and AI might be sufficiently large and excessively correlated), and in such a case, even optimal reliance decisions by human DMs would not result in complementarity. One way to identify any successes or failures of complementarity is to observe the performance differences in a paradigm in which the human DM makes the final decision and a paradigm in which the independent human and AI decisions are statistically combined into a final decision. However, relying on an external statistical aggregator to identify and leverage complementarity of the AI is not a viable solution in cases in which the human DM makes the final decision. As we discuss regarding Challenge 2, it is important to empower human DMs to build appropriate mental models of their AI assistants so they may leverage the complementary ability of the AI. Does the human make effective use of the information that is made available by the AI?

## Improving Human–AI Complementarity

Additional research must be conducted to better understand the factors that contribute to human–AI complementarity and to develop new methods for promoting complementarity. In the context of AI research, new AI systems are developed to take into account the fact that a human is part of the decision-making process (Bansal, Nushi, et al., 2021; De et al., 2020; Wilder et al., 2021). These AI systems are trained to optimize for the joint performance that can be expected when the human leverages the AI to facilitate decision-making. In the context of psychology, additional research is necessary to understand the ways in which the degree of independence of AI predictions affects human decision-making. In human teams, some degree of cognitive diversity among members of the group contributes positively to team performance, but researchers have hypothesized that excessive cognitive diversity might negatively affect communication among team members and thus lead to suboptimal team performance (Aggarwal et al., 2015). Likewise, whereas independence between human and AI predictions contributes to complementarity, AI predictions that are excessively different from human predictions might not be perceived as useful (Grgić-Hlača et al., 2022). Therefore, additional research is necessary to understand the psychological limitations that might prevent human DMs from making effective use of AI predictions.

## Challenge 2: Understanding Human Mental Models of AI

An important determinant of the effective use of AI assistance is the human mental model of the AI in question, which contains a person’s collection of beliefs regarding the AI and expectations concerning the effects of interacting with the AI. In general, mental models are simplified representations of the world that are constructed by humans to allow them to integrate new information and make predictions while expending little mental effort (Craik, 1952; Smyth et al., 1994). Hence, the more accurate the mental model of the AI is, the more likely it is for the AI to be used correctly (Bansal et al., 2019). Likewise, ineffective use of AI might be driven by incomplete and/or incorrect mental models of the AI. Such incorrect mental models may lead to inappropriate levels of reliance on or miscalibrated trust in the AI. We posit that a deeper understanding of people’s mental models of AI can facilitate the design of workflows that can aid humans in developing appropriate reliance strategies and consequently lead to improved team performance.

Studies on people’s mental models of AI have indicated a wide range of conceptions about AI. To organize and understand these empirical results, we distinguish between mental models of AI that are developed before people have actually experienced the AI in question, in which the mental model is driven mostly by prior beliefs, and how these models compare with the models that humans build of other humans. We also discuss how people’s mental models of AI are shaped by experiences of interaction with the AI.

Several studies have investigated people’s prior beliefs, in which participants have been asked how they would use AI advice relative to advice from a human in various hypothetical scenarios. The results are strongly dependent on the way in which the scenario is framed, including the task domain, the amount of information that is provided regarding AI performance, and individual differences (Abraham et al., 2017; Bigman & Gray, 2018; Castelo et al., 2019; Lubars & Tan, 2019). When given the choice, people prefer to rely on humans than on AI in highly consequential scenarios (Castelo et al., 2019), especially in the context of hypothetical moral scenarios in which life and death hang in the balance (Bigman & Gray, 2018). For tasks associated with a high perceived degree of objectivity (e.g., those that involve quantifiable facts vs. personal opinions and intuitions), this preference for humans is transformed into a preference for AI (Castelo et al., 2019). In the context of several low-stakes quantitative tasks, such as estimating the weight of a person from a photograph or predicting the popularity of songs, people actually prefer to take advice from algorithms than from other humans (Logg et al., 2019). In addition, people’s preferences for relying on AI become stronger when performance data regarding the AI are provided (Castelo et al., 2019). The willingness of people to consider the use of automation also depends on demographic factors. For example, younger users are more willing to use automation in vehicles (Abraham et al., 2017). Understanding these preferences regarding task delegability and expectations of AI performance is important because these factors might affect people’s willingness to accept AI advice when they actually interact with AI decision-support systems.

Another set of studies has investigated people’s mental models of a particular AI after their initial exposure to the AI. At first glance, these experiments have seemed to present a mixed picture of people’s understanding of AI and the effectiveness of their reliance decisions. For example, Dietvorst et al. (2015) showed that participants prefer to rely on human decision-making than on algorithms after witnessing the performance of an algorithm, which includes cases in which the algorithms make mistakes, despite the fact that the algorithm actually outperforms the human DMs on average. This result has been viewed as suggesting that experience with the AI, especially exposure to errors made by the AI, leads to “algorithm aversion,” presumably because people expect algorithms to perform better than they actually do (e.g., for an overview, see Burton et al., 2020). However, these experimental studies have faced an important limitation. While individuals became familiar with the algorithm’s performance, they were asked to make a delegation decision only once, and they were not informed of the consequences of that delegation decision. Therefore, these results cannot be used to answer questions regarding whether humans use AI advice selectively.

In contrast, in recent studies (M. Kelly et al., 2023; Liang et al., 2022; Tejeda et al., 2022), participants have been provided with numerous opportunities to make reliance decisions, and the DM has been allowed to selectively use algorithmic advice. These experiments have not confirmed claims regarding people’s general algorithm aversion. Instead, the results reported by Tejeda et al. (2022) show that participants adopt a flexible reliance strategy according to which reliance depends on the DMs’ own confidence state, the confidence expressed by the AI, and overall AI performance. In addition, these results showed that this reliance strategy is effective and does not differ substantially from optimal reliance strategies. Other studies have found that people can take into account the accuracy of algorithmic advice (Liang et al., 2022; Yin et al., 2019). Remarkably, individuals can calibrate their dependence on AI even in the absence of explicit accuracy feedback (Lu & Yin, 2021; Wang et al., 2022). They can achieve this by using instances in which they possess high confidence in their own performance to evaluate the capabilities of AI or other individuals (Pescetelli & Yeung, 2021). Furthermore, a recent study conducted by M. Kelly et al. (2023) examined the comparative assessments made by individuals regarding the abilities of AI systems and other humans in trivia-related tasks. The findings suggest that individuals’ appraisal of others is profoundly shaped by their own perceived capacities. This correlation does not extend to evaluations of AI; individuals’ assessments of AI significantly diverge from their self-appraisals. Furthermore, there is a prevalent expectation among individuals that AI agents will outperform humans across diverse trivia categories.

Overall, these empirical results have suggested that people’s mental models of AI depend on their degree of familiarity with the AI in question and their degree of familiarity with the outcomes of their reliance decisions. It is possible that people who are somewhat familiar with the AI’s performance but not with the consequences of their own decisions to delegate or rely on AI advice might have an incomplete mental model and might not represent the differential capabilities of AI relative to themselves accurately. Perhaps their mental assessment of the AI is (correctly) downgraded after exposure to inevitable AI errors but does not correctly reflect the fact that they themselves might not fare any better when attempting to solve the same problem and, in fact, that they might perform even worse in that context. However, the results of studies in which people were informed of the consequences of their reliance decisions suggest that people develop richer mental models of AI that allow for flexibility with respect to relying on their own decisions or those of the AI. Other factors, such as the complexity of the AI and the decision-making task at hand, likely affect mental-model fidelity as well. Some laboratory tasks focus on relatively simple behavioral tasks that might not require a great deal of learning to develop effective reliance strategies. However, in the context of complex industrial systems or military applications associated with higher levels of automation, the DM might not fully understand how the system works and might thus default to simplistic strategies such as indiscriminately relying on the AI (Cummings, 2017).

## Improving the Assessment of Mental Models

Understanding people’s mental models of AI requires new research in several directions. First, little is known at the moment regarding long-term changes in human beliefs about AI (Glikson & Woolley, 2020). Longitudinal studies must be conducted to understand the changes in people’s mental models over time. Do these mental models become more accurate over time? In addition, methods such as cognitive modeling can be used to make inferences regarding the latent content of people’s mental models, including their decision-making strategies and beliefs that cannot be directly assessed using behavioral measures (e.g., Chong et al., 2022; Tejeda et al., 2022). Given that humans’ mental models of interaction with the AI encode perceived differences between their own capabilities and those of the AI, it could be useful to leverage insights drawn from psychological research on metacognition to understand the ways in which people estimate their own self-confidence (Koriat & Levy-Sadot, 1999) and their performance relative to others (Moore & Cain, 2007). In addition, it is plausible that individuals’ cooperation with AI is guided by straightforward learning approaches, such as model-free reinforcement learning, rather than by explicit mentalization of the AI assistant’s abilities. Further research is required to determine whether individuals develop explicit representations of the AI or rely on basic heuristics when integrating its recommendations.

## Challenge 3: Developing Effective Methods of Interaction With AI

The task of developing accurate mental models of AI is crucial to effective and efficient human-AI collaboration. Hence, it is critical to develop workflows and systems that aid human DMs in constructing accurate mental models of their AI teammates. Specifically, we consider two major design choices that influence the ways in which AI assistance is used by human DMs: the choice of when to present AI assistance and the choice of what information to present. In addition, we discuss adaptive methods that can tailor AI output and human-AI interaction to take human cognitive limitations into account.

### When should AI assistance be presented to the human DM?

Several studies have investigated the impact of presenting AI assistance at different times throughout the decision-making process. These manipulations have been designed to increase people’s cognitive motivation to engage with the AI assistant’s recommendations and explanations (Buçinca et al., 2021). We categorize these advice presentation paradigms into the following groups: (a) concurrent, (b) sequential, (c) on demand, and (d) time delayed.

In concurrent paradigms, when the problem is introduced, the AI advice is shown to the DM immediately. In the sequential paradigm, which is known in the decision sciences as the “Judge Advisor System” (Bonaccio & Dalal, 2006), AI advice is shown only after the DM first makes an independent decision. After receiving advice from the AI, the DM is then presented with the opportunity to update their decision. Some research suggests that the sequential paradigm improves AI-assisted decision accuracy compared with humans’ accuracy when doing the task without assistance (Green & Chen, 2019), presumably because it encourages independent reflection, which could lead to the retrieval of additional problem-relevant information. However, other studies have found no differences in overall performance in this context (Buçinca et al., 2021; Tejeda et al., 2022).

The on-demand paradigm allows DMs to selectively seek out AI assistance (Buçinca et al., 2021; Kumar et al., 2021; Liang et al., 2022). This approach requires DMs to engage in a metacognitive process that involves assessing the expertise of the AI assistant relative to themselves and seeking its help. Note that the on-demand paradigm is a variation of the sequential paradigm because both encourage the DM to make an initial judgment before receiving the AI assistant’s advice. Kumar et al. (2021) proposed a computational model of this metacognitive decision to seek help. Buçinca et al. (2021) compared team performance directly between the on-demand paradigm and the sequential and time-delayed paradigms. Although these authors did not find any improvement in overall accuracy, additional studies must be conducted to better understand decision-making in this paradigm. Finally, the time-delayed paradigm delays the provision of AI advice, which can improve decision accuracy (Park et al., 2019). One explanation for this effect is that the delay offers DMs additional time to reflect on the problem and improve their own decision-making, thus reducing the anchoring effect. Another approach is to vary the amount of time that is available for people to process the AI prediction, which is shown immediately (Rastogi et al., 2022). Rastogi et al. (2022) showed that people are more likely to detect AI errors when more time is made available. Further investigation is necessary to understand the effects of the time at which the AI advice is provided.

### What information should the AI present to the human DM?

AI-assisted decision-making is a form of advice taking in which the human may take advice from an AI assistant. The human-human advice-taking literature has indicated that humans tend to discount advice from others because they do not have access to others’ reasoning (Bonaccio & Dalal, 2006; Gino & Moore, 2007). Working with an AI is no different. Research has shown that humans are susceptible to a variety of misjudgments and biases when taking advice from AI (Logg, 2017; Logg et al., 2019). Hence, the task of developing interpretable and explainable AI that can make the process by which the AI generates advice apparent is key to establishing a productive working relationship between humans and AI.

#### AI confidence

Most AI systems can calculate confidence measures for their predictions, such as confidence intervals for regression tasks or estimated probabilities of accurate predictions in classification tasks (Bhatt et al., 2021). These confidence measures assist DMs in calibrating their mental models of the AI and determining when it may make incorrect predictions (Bansal et al., 2019; Y. Zhang et al., 2020). DMs are more inclined to adopt solutions with high AI confidence than those with low AI confidence (Y. Zhang et al., 2020). Furthermore, Tejeda et al. (2022) found that the confidence differential between AI and DM influences reliance decisions. DMs are more likely to follow the AI’s advice if the AI demonstrates high confidence while the DMs, based on their independent decision-making process, exhibit low confidence.

#### AI explanations

A variety of techniques have been developed to augment AI predictions with explanations. One such type of explanation is the identification of the set of features that contributes to the model prediction (Lakkaraju et al., 2022). This supplementary information can be valuable because it allows DMs to discern when AI predictions are based on reasonable or flawed reasoning, thus allowing them to modulate their reliance on the AI system accordingly. However, mixed evidence has been reported regarding the helpfulness of feature-attribution methods (Bansal, Wu, et al., 2021; Buçinca et al., 2021; Chen et al., 2022; Nguyen et al., 2021). For example, Bansal, Wu, et al. (2021) showed that providing feature attributions did not improve human-AI complementary performance over a baseline condition in which the AI indicated only its level of confidence. In addition, these types of explanations merely increased people’s tendency to adopt AI advice regardless of its correctness. Despite the potential shortcomings of feature attributions, alternative methods exist for presenting AI explanations. One notable approach involves showcasing a set of examples that the AI model perceives as similar to the target problem along with demonstrating how these features interrelate across these examples. This method can assist human DMs in determining whether the AI is referencing an appropriate class and if it has accurately recognized the relationships between features (Nguyen et al., 2021; Taesiri et al., 2022).

One potential risk of providing AI explanations is that people might process the explanations only superficially and might use the presence of such explanations as a heuristic for model accuracy. Therefore, designing behavioral interventions that increase cognitive effort and analytic thinking might make it more likely for people to use explanations effectively (Buçinca et al., 2021). To increase the cognitive engagement of the DM, Gajos and Mamykina (2022) argued that it might be beneficial to show only the AI’s explanation (i.e., to highlight information relevant to the problem) and to withhold the AI’s prediction.

Generally, explanations must be designed to take the human user into account (Hoffman et al., 2018). Instead of designing explanations to be convincing, which leads to inappropriate reliance, it is better to design explanations to be as informative as possible (Bansal, Wu, et al., 2021). As Lee and See (2004) noted, the objective of this process is not to design systems to increase reliance or trust but to design them to produce appropriate levels of reliance and trust.

### Toward adaptive and interactive AI assistance

Overall, empirical evidence has demonstrated that providing more information regarding the AI does not always increase performance. Given the limited cognitive resources that might be available to process AI recommendations, especially in time-sensitive (time-poor) situations, it is important for the AI to adjust its output (e.g., by providing explanations at the right level of detail). Excessive information can be detrimental to decision-making (Poursabzi-Sangdeh et al., 2021; Schaffer et al., 2019). Therefore, AI systems must be designed to adapt to the cognitive limitations of the human DM (Cummings, 2017). The questions of what, when, and how much information should be presented to a human DM highlight the need to develop theoretical frameworks that infer the impact of AI aids on human cognition and observed performance. Such frameworks are now starting to emerge in the context of explainable AI (Chen et al., 2022). In addition, theories and computational models drawn from psychology can be leveraged to better understand human cognition when collaborating with an AI (Rastogi et al., 2022). For example, in situations in which decisions must be made quickly or in which varying degrees of mental effort are required to process the AI’s output, theories of rational resource allocation (Gershman et al., 2015; Lewis et al., 2014; Lieder et al., 2018; Lieder & Griffiths, 2020) could be used to identify when people might disregard AI predictions if the perceived gains do not warrant the associated costs in terms of time and mental effort.

Research in psychology and behavioral economics has long advocated for interventions or “nudges” to steer people’s decision-making (Thaler & Sunstein, 2008). Advances in computational theories of nudging (Callaway, Hardy, & Griffiths, 2022; Callaway, Jain, et al., 2022) have enabled the identification of optimal decision strategies and effective feedback to guide decision-making. Callaway, Jain, et al. (2022) demonstrated that AI assistants can successfully prompt people to adopt optimal decision strategies when given feedback about their decision-making process. In a similar vein, optimal gamification can redesign the reward structure of the environment to align people’s long-term goals with their short-term rewards. This approach helps people overcome myopic decision-making tendencies and behave in a more far-sighted manner when faced with sequential tasks (Consul et al., 2022; Lieder et al., 2019). AI-aided nudging is a powerful paradigm of adaptive AI assistance that can be tailored to people’s abilities and can reduce cognitive load of long-term optimization.

Finally, another promising research direction is to make the AI output more interactive. Instead of presenting explanations in the form of one-off outputs, Lakkaraju et al. (2022) argued for the importance of interactive AI systems. In these systems, the human DM can query the model concerning why a decision was made using natural-language dialogue that allows the AI prediction to be clarified through a series of interactions. Moreover, interactive AI assistants have been shown to improve user acceptance and trust. For example, pathologists reported increased diagnostic utility and higher trust when they were able to customize their search in content-based image-retrieval systems (Cai et al., 2019). Allowing for communication and interaction with AI assistants can improve people’s understanding of the system.

## Discussion and Conclusion

Although the body of empirical research on AI-assisted decision-making continues to expand, there is still much to uncover about the potential of human–AI collaboration. This is because numerous laboratory studies have faced limitations in various aspects. First, many empirical studies have involved algorithmic advice from simulated AIs or “wizard-of-Oz” procedures (Lai et al., 2021) that produce decisions at preset levels of accuracy and agreement with the human (e.g., Gajos & Mamykina, 2022; Grgić-Hlača et al., 2022; Liang et al., 2022; Pescetelli et al., 2021). Although this approach simplifies the process of conducting behavioral studies, it omits an important aspect of actual AI systems—the fact that they are correlated to some degree with human decision-making (Steyvers et al., 2022; Tejeda et al., 2022). Second, many laboratory studies have involved low-stakes decision problems that require relatively little expertise from participants. It is not entirely clear how the results of these empirical studies will generalize to more complex and high-stakes contexts, such as healthcare decisions (Buçinca et al., 2020; C. J. Kelly et al., 2019). Finally, most empirical studies have focused on only a limited temporal window to assess human understanding of AI. In extreme cases, some studies have involved single-shot AI-reliance decisions (e.g., Dietvorst et al., 2015) that might not provide a complete picture of selective human reliance on AI. Other empirical studies have often been limited to single experimental sessions and have not provided insights into long-term changes in human beliefs regarding AI or changes in AI-reliance strategies (Glikson & Woolley, 2020; Nishikawa & Bae, 2018).

In the context of the long-term use of AI decision aids, additional factors may have to be considered. For example, theories of human motivation, such as self-determination theory (Ryan & Deci, 2000), have emphasized the importance of perceived autonomy because the sense of control and agency can improve overall engagement and outcomes. If a human DM perceives a lack of autonomy, this might negatively affect engagement and ultimately lead to worse outcomes. For example, Dietvorst et al. (2018) showed that allowing individuals to intervene in an algorithm’s decision caused the individuals in question to appreciate the algorithm more and made them more likely to use the algorithm in the future even though allowing the algorithm to make the decision without intervention would lead to better outcomes. The authors argued that allowing humans to have some degree of control ultimately might lead to better overall outcomes and avoid a situation in which humans avoid using algorithms altogether. In the framework of AI-assisted decision-making, the final decision lies with the human, and therefore, one could argue that this approach leaves ample room for human autonomy. However, many potential factors associated with this framework could affect the perceived control of the decision, which could in turn influence the human’s willingness to rely on the AI (Chugunova & Sele, 2022). For example, the AI might not reveal its predictions but merely show information that is relevant to the prediction to increase human engagement and perceived agency (Lai & Tan, 2019). In general, more research is necessary to understand the ways in which offering human DMs additional control over the AI (e.g., allowing them to determine the extent of AI explanation) affects performance because this approach might increase perceived agency but also lead to cognitive overload and ineffective use of the available information.

To gain a deeper understanding of how humans depend on AI, researchers can build on prior work on human-to-human advice that aims to elucidate the strategies individuals employ to integrate the opinions of others when reassessing and refining their own judgments (Himmelstein, 2022; Jayles et al., 2017). Analogous to studies on AI-assisted decision-making, these studies share common themes. Both areas of research are interested in understanding how advice is elicited, whether from human or AI sources. This includes the communication and presentation of advice and how the credibility of the advice source influences the likelihood of it being accepted. Furthermore, in both cases, researchers are interested in understanding how people aggregate multiple opinions to form a revised judgment. This includes exploring the weight individuals assign to different sources of advice and the strategies used to combine the information. However, there are also notable differences when comparing human and AI advice. AI-generated advice may be more difficult to understand or interpret, especially when complex algorithms are involved. This may affect how people incorporate AI advice compared with human advice, which is typically more transparent and easier to relate to. Human advice may also be influenced by emotional and social factors, such as empathy, and personal relationships. These factors can play a role in the acceptance of human advice but are usually absent in AI-generated advice. Finally, the use of AI-generated advice raises ethical questions, such as algorithmic fairness and the potential for biased recommendations. Whereas human advice can also be biased, the ethical implications of AI advice might differ, leading to unique challenges in this area of research. Overall, although research on humans taking advice from other humans and humans taking advice from AI share several common themes, the unique aspects of AI-generated advice introduce new dimensions to the decision-making process that warrant further exploration.

Although researchers can also draw insights from an extensive body of research on human reliance on simple algorithmic decision aids (Arkes et al., 1986) and automation (Parasuraman & Riley, 1997), we argue that several distinct factors come into play when AI serves as an assistant rather than a decision aid grounded in a straightforward algorithm. AI is capable of offering a diverse range of information beyond mere recommendations, such as confidence levels and detailed explanations, while also possessing the ability to adapt to the cognitive constraints of the human DM. This permits the conceptualization of entirely new workflows that can reduce the workload of the human. For example, instead of the human and the AI collaborating on individual problems, they might instead collaborate to define the rules that delineate the full set of problems into types that the human DM should address and those for which the AI is very likely to exhibit good performance (Lai et al., 2022). Note that in this workflow, the human DM relinquishes control over some decision problems but retains high-level control with respect to defining the types of problems that are addressed by the AI.

To truly add value in the context of assisting humans, it is necessary to develop AI systems that can go beyond providing superficial assistance and instead serve as useful teammates to a human DM. One way to accomplish this task is to enable the AI to develop mental models of the human DM that can allow the AI to infer the mental states, expertise, workload, long- and short-term goals, and beliefs of the human DM. We posit that computational cognitive modeling must play a critical role in developing such AI assistants. Cognitive modeling enables researchers to model the latent cognitive states of humans and make predictions regarding their future actions, beliefs, and knowledge states. Most previous work in the cognitive-science literature has focused on constructing models of decision-making when humans work in isolation or with other humans (e.g., Himmelstein, 2022). There has been a recent surge of interest in the development of computational models for the human’s decision-making process with the assistance of an AI (see Kumar et al., 2021; Oulasvirta et al., 2022; Tejeda et al., 2022). These models serve as approximations of the human DM’s mental processes and may be used to develop AI assistants that are sensitive to the cognitive states of human DMs. Although enabling AI to build explicit mental models of human DMs is a key path to building helpful AI assistants, in some cases, it may be sufficient for AI assistants to rely on heuristics that are grounded in the principles of cognitive science. For example, an AI assistant may cleverly display only the most relevant insights for every task to the human to avoid overwhelming the DM with excessive information.

The continuous evaluation of AI-assisted decision-making is essential to ensure improved performance. Current evaluations are based on simple empirical metrics such as overall accuracy and the turnaround time of decisions. However, these metrics provide only a limited view of the decision-making process. It is necessary to understand and quantify what it means for the AI to be truly helpful in further detail. The first challenge prompts researchers to pay close attention to the development of AI that can complement human abilities and provide useful assistance. In addition, it may be useful for the AI assistant to infer the human’s understanding of the problem at hand and the human’s understanding of the AI assistant’s decision-making process. The second challenge emphasizes the need to understand the human’s mental models and develop adaptive AI assistants. Finally, the third challenge calls for the careful design of human–AI interactions. These design decisions must be guided by the latent cognitive states of the human DM.

In conclusion, to optimize AI-assisted decision-making, scientists must venture beyond merely improving the AI or developing better methods of improving human decision-making to consider a multitude of factors that are related to the human, the AI assistant, and the interaction between the human and the AI. The three challenges discussed in this article will remain pertinent as AI assistance becomes more prevalent. Ultimately, AI systems have the potential to play more than the simple role of a static information provider and can be designed to pursue more general objectives, such as assisting a human to the greatest extent possible (Russell, 2019) and learning to complement the human DM’s expertise (Wilder et al., 2020). Answering the question of how to design and quantify such general objectives requires an interdisciplinary approach that combines insights from several disciplines, including AI and machine learning, human–computer interaction, and psychology.

## Transparency

*Action Editor:* Sudeep Bhatia

*Editor:* Interim Editorial Panel
